# Imported allergens in Italy: an emerging issue

**DOI:** 10.1186/s13052-024-01595-z

**Published:** 2024-03-03

**Authors:** Luca Pecoraro, Mattia Giovannini, Francesca Mori, Simona Barni, Riccardo Castagnoli, Stefania Arasi, Carla Mastrorilli, Francesca Saretta, Lucia Liotti, Lucia Caminiti, Angela Klain, Mariannita Gelsomino, Michele Miraglia Del Giudice, Elio Novembre

**Affiliations:** 1 Pediatric Unit, Department of Surgical Sciences, Dentistry, Gynecology and Pediatrics, University of Verona, 37126 Verona, Italy; 2 Allergy Unit, Meyer Children’s Hospital IRCCS, 50139 Florence, Italy; 3 Department of Health Sciences, University of Florence, 50139 Florence, Italy; 4 Department of Clinical, Surgical, Diagnostic and Pediatric Sciences, University of Pavia, 27100 Pavia, Italy; 5 Pediatric Clinic, Fondazione IRCCS Policlinico San Matteo, 27100 Pavia, Italy; 6 Translational Research in Pediatric Specialties Area, Division of Allergy, Bambino Gesù Children’s Hospital [IRCCS], 00165 Rome, Italy; 7 Pediatric Hospital Giovanni XXIII, Pediatric and Emergency Department, AOU Policlinic of Bari, 70126 Bari, Italy; 8 Pediatric Department, Latisana-Palmanova Hospital, Azienda Sanitaria Universitaria Friuli Centrale, 33100 Udine, Italy; 9 Pediatric Unit, Department of Mother and Child Health, Salesi Children’s Hospital, 60123 Ancona, Italy; 10 Department of Human Pathology in Adult and Development Age “Gaetano Barresi”, Allergy Unit, Department of Pediatrics, AOU Policlinico Gaetano Martino, 98124 Messina, Italy; 11 Department of Woman, Child and of General and Specialized Surgery, University of Campania ’Luigi Vanvitelli’, Naples, Italy; 12 Allergy Unit, Pediatrics Area, Department of Woman and Child Health, Policlinico Gemelli University Foundation IRCCS, Catholic University of Sacre Hearth, 00168 Rome, Italy

**Keywords:** Imported allergens, Ambrosia artemisiifolia, Cannabis sativa, Ethnic food, Asian Wasp Velutina

## Abstract

Imported allergens are involved in many allergic reactions, with unexpected and unusual implications. They can be involved in developing asthma, allergic rhinoconjunctivitis, Hymenoptera venom allergies and food allergies. Imported allergens can be implied in respiratory allergies attributable to commercial practices and accidental diffusion through air currents that have introduced non-native species in new geographical contexts. *Ambrosia artemisiifolia L*., a plant native to North America and currently in the western part of Lombardy, represents an example. Moreover, a variation in the pollen concentration in the Northwest Tuscany area and Trentino Alto-Adige was observed. *Cannabis sativa* is another imported allergen used frequently by adolescents. Regarding potential imported food allergens, there is no validated list. Imported food allergens derive from ethnic foods, referring to Mexican/Latin American, Chinese/Japanese, Southeast Asian, Arab/Middle Eastern and African cuisine. Four insect flours were recently introduced to the European and Italian markets (*Acheta domesticus, Alphitobius diaperinus, Tenebrio molitor* and *Locusta migratoria*). The association between the accidental introduction through commercial traffic, climate change, and the absence of natural enemies in the destination ecosystem is related to the introduction of a specific Hymenoptera, *Vespa velutina*, in Italy and Europe. External events attributable to human activities, such as climate change and the introduction of non-native plants, foods and Hymenoptera through trade, have contributed to the issue of imported allergens. Making the correct diagnosis and guiding the diagnostic and therapeutic path in this particular context represent the concerns of the pediatric allergist.

**Supplementary Information:**

The online version contains supplementary material available at 10.1186/s13052-024-01595-z.

## Introduction

Allergy is an immune reaction to common environmental allergens [1]. Common allergens include pollens, fungal spores, house dust mites, animal epithelium, foods, biological products, and Hymenoptera venom [2]. At the same time, potentially all environmental substances can act as allergens and cause an allergic reaction [3]. Thus, “imported” allergens from foreign countries may be involved in allergic reactions with unusual and unexpected connotations [3]. Specifically, they may be involved in the pathogenesis of allergic rhinoconjunctivitis, asthma, food allergy, and Hymenoptera venom allergy.

## Respiratory allergy

Respiratory allergies caused by imported allergens can be attributed to air currents and trade practices that introduced non-native species to the new geographic setting [4]. Once a plant seed is introduced to a new area, multiple factors are involved in the spread of its pollen allergen, such as urbanization and climate change. These factors can affect both local and imported pollen’s timing, quantity, and allergenicity. Specifically, higher carbon dioxide concentrations and temperatures can increase pollen and induce longer pollen seasons. There is evidence that pollen allergenicity can increase as a result of both climate change and interaction with air pollutants [5, 6]. One example is *Ambrosia artemisiifolia L.* (common ragweed), a plant native to North America that has developed in Europe in recent decades [7]. In Italy, it is currently found in the western part of Lombardy [8]. Ragweed is a plant that prefers a temperate climate and proliferates in dry, sunny, grassy areas in sandy soils, riverbanks, roadsides, and abandoned fields [9]. Generally, ragweed requires a warm climate to take root and release pollen [10]. Climatically, the Mediterranean area seems only suitable for the rooting and survival of ragweed, not favoring its flowering. However, ragweed’s ease of growth, absence of natural enemies, resistance to herbicides, and the high genetic variability of invasive populations mean that in some countries, such as the Netherlands, Belgium, and the Mediterranean, ragweed pollens are present in greater quantities than expected [11–14]. In Italy, ragweed was first reported in 1901 in Piedmont, arriving in Lombardy in the 1940s and spreading consistently since the 1980s. Currently, the northwestern area of Milan and the southern area of Varese are where ragweed is most prevalent [15]. Ragweed pollen is extremely allergenic and can produce 100 million to 3 billion pollen grains [16]. Symptoms of rhinitis and asthma may develop in ragweed-allergic individuals [17]. Treatment strategies are superimposed on those for pollen-induced respiratory allergies: allergy avoidance, medical therapy, and allergen-specific immunotherapy [18]. In the presence of high CO2 levels, ragweed produces more pollen. At the same time, climate change leads to an increase in the pollination period of ragweed. Considering these factors, ragweed pollen production may increase significantly in the future, as may its impact on allergic rhinoconjunctivitis and asthma [19, 20]. An additional example of the impact of climate change on the environment is the change in prevalent mold and pollen concentrations in northwest Tuscany from 2010 to 2019. Specifically, an upward trend was found for ragweed, Alternaria spores, and grasses (the latter in the summer and fall). In contrast, a decreasing trend was observed for birch and Cupressaceae. No differences were found inherent in the duration of pollen seasons and the timing of pollen initiation and termination. Increased environmental temperatures and humidity favor the proliferation and spread of ragweed, Alternaria spores, and grasses. At the same time, they hinder the flowering of birch and *Cupressaceae* [21]. Climate change also seems to be involved in the increase in mugwort pollen concentrations in Trentino-South Tyrol. This occurrence also seems to be due to the increased spread of two specific invasive allochthonous types, *Artemisia annua* and *Artemisia verlotiorum*, which may threaten the biodiversity of native plants. Allergic symptoms occur in September; the increased concentration of such pollen can exacerbate allergic symptoms in affected individuals [22]. Cannabis is another example of an imported allergen that also assumes relevance in the pediatric setting, given its frequent use in adolescents [23–24]. *Cannabis sativa* is a plant native to Asia that flowers from late summer to early fall. The importation and, therefore, the use of Cannabis sativa has increased compared to the past decades; at the same time, an increased incidence of cannabis allergy has been observed [25]. In Italy, the Ministry of Health regulates its dispensation to patients, regardless of the acquisition procedure (through duly authorized companies or the ministerial authorization procedure - DM February 11, 1997), which must be done as a magistral preparation on a nonrepeatable prescription from the treating physician, drawn up in accordance with the provisions of Law 94/98. *Cannabis sativa* is the most widely used recreational drug in the world, and its illegal cultivation is widespread [26]. Cannabis pollen, similar to other pollens, is capable of causing allergic reactions by inhalation. The flowering period in outdoor cultivation usually begins in mid-July and lasts 6–8 weeks. In indoor cultivations, the flowering period is dependent on light exposure and begins when light cycles are set with 10–12 h of darkness. Potential clinical manifestations associated with exposure to cannabis pollen include rhinitis, conjunctivitis, contact urticaria, asthma, and in rare cases, even anaphylactic shock [26]. In fact, the hemp seeds from which cannabis is derived can be ingested, giving symptoms of food allergy, while contact with cannabis dust can cause symptoms of an occupational cannabis allergy, such as contact dermatitis and asthma [27–29]. The pathogenesis of allergic reactions to cannabis is related to exposure to allergens specific to Cannabis sativa and reactions to cross-reactive allergens with structurally similar plant foods. Specifically, cannabis contains an allergen, Can s 3, which belongs to the non-specific lipid transfer protein (ns-LTP) and is present in vegetables and fruits, including peaches, apples, tomatoes, eggplant, chestnuts, almonds, and walnuts. In individuals sensitized to LTP from cannabis, cross-reactivity reactions can occur with LTP present in fruits and vegetables due to a cross-reactivity mechanism. This syndrome is called “cannabis-fruit-vegetable syndrome” [30]. In addition, cross-reactive allergens have been shown to be present in cereals, tobacco, latex, wine, and beer [30]. A diagnosis of allergy to *Cannabis sativa* is based on the patient’s history, which is often not easy to collect due to the illegal use of this substance, and on a skin prick test, extracts of which are usually set up from crushed buds, leaves, and flowers of the plant [25, 26]. The lack of commercial extracts and standardized and validated in vitro tests do not allow an adequate diagnostic work-up of cannabis allergy. Treatment does not differ from that of other forms of respiratory or food allergies. Sporadic cases of intramuscular or subcutaneous specific allergen immunotherapy with Cannabis sativa have also been reported to date without solid demonstration of efficacy [25].

## Food allergy

**Fig. 1:**
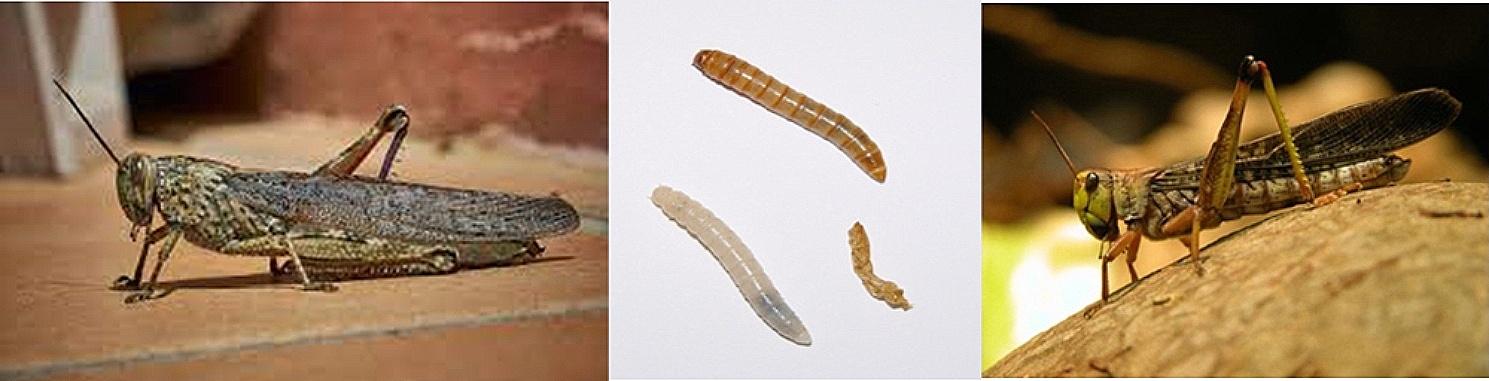
Examples of edible insects (cricket, mealworm, grasshopper)

Food allergy caused by imported food allergens is an emerging problem, as 84.7% of the Italian population have consumed ethnic food at least once in their lifetime [31]. At present, there is no validated list of potential imported food allergens. One can infer their consumption in Italy through purposefully collected case histories. Specifically, the most commonly consumed ethnic foods come from the cuisine of Chinese or Japanese (38.8%), followed by Mexican/Latin American (25.7%), Arab/Middle Eastern (14.2%), Southeast Asian (10.6%), and African (5.4%) [31]. Japanese and Chinese cuisines mainly use peanuts, fish, shellfish, soy, and eggs. Regarding soy sauce, which is always present in Asian restaurants, it is generally well tolerated by those with soy allergies because soy proteins are destroyed by the fermentation process [32, 33]. The so-called “Chinese restaurant syndrome” is controversial; it is characterized by pressure on the face, chest pain, burning sensations throughout the body, and anxiety and is due to the ingestion of MSG, which is used as a food additive in many Chinese dishes [34]. The exact etiology of this syndrome is unknown, but studies in guinea pigs have demonstrated the neurotoxic and neuroexcitatory properties of MSG in the hypothalamic region of the central nervous system [35]. In any case, the analysis of case histories reported in the literature does not confirm a causal relationship between MSG ingestion and patient symptoms [36]. Mexican cuisine uses sauces containing multiple allergens, such as cocoa, spices, and nuts. Also frequent is the use of beans and spirits containing traces of soy or wheat [37]. In contrast, Middle Eastern cuisine is characterized by spices, olive oil, nuts, and oilseeds (including tahini) [38]. Spices, legumes, and nuts are also present in Southeast Asian cuisine. Furthermore, in particular types of tea or curries, one must check for the possible presence of cow’s milk [38]. Regarding allergens, African cuisine uses peanuts to a great extent [39]. Recently, insect meal has been allowed to enter the European market and, therefore, Italy [40]. Specifically, the European Union has authorized the use of insects in food products since 2018 [41]. In Italy, four insect meals were authorized for consumption in March 2023: *Acheta domesticus* (house cricket), *Tenebrio molitor* (yellow grub of meal), *Alphitobius diaperinus* (lesser meal worm), *Locusta migratoria* [42]. Nutritionally, insects contain a large amount of protein, possess high nutritional value, and have antioxidant, anti-inflammatory, anti-adipogenic, and antidiabetic power [43]. Therefore, insects are considered a healthier nutritional source than red meat. Their inclusion in the Mediterranean diet can reduce risk factors for some diseases, such as diabetes, obesity, and hypertension. In addition, their farming reduces environmental impact since much less CO2 is generated than in farmed meat production [44]. As edible insects have been introduced into the diet, possible food allergies to these insects have arisen [45]. However, there is a substantial lack of information regarding the allergenicity of edible insects and the symptomatology by which allergic reactions occur [46]. Food allergy to insects has been described for silkworms, mealworms, caterpillars, *Bruchus lentis*, sago worms, locusts, grasshoppers, cicadas, bees, *Clanis bilineata*, and the food additive carmine, which is derived from the females of the insects *Dactylopius coccus* (Fig. 1) [45]. Only inhalation allergy studies have been described for cockroaches, which are also edible insects [45]. Several insect allergens, including tropomyosin and arginine kinase, pan-allergens with cross-reactivity with homologous proteins, such as crustaceans, mollusks, and house dust mites, have been identified. Cross-reactivity and/or co-sensitization of insect tropomyosin and arginine kinase have been demonstrated in allergic patients [45]. Currently, there are no prevalence studies on this food allergy, even in Southeast Asia and China, where insects are routinely consumed. Despite the absence of significant case histories, the prevalence of insect allergy does not appear to be high, as several studies conducted in Asia (including China and Thailand) on the prevalence of food allergy do not report insects as a frequent cause of food allergy [47, 48]. In a study conducted in Laos and based on a questionnaire, out of 1059 adult subjects who had previously eaten insects, 81 (7.6%) reported that they had experienced an allergic reaction from eating insects. The type of insects was not specified, however, and no cases of severe anaphylaxis were reported [49]. Severe anaphylactic reactions from insect ingestion, however, have been reported in other studies: In a review collection of 358 cases of severe anaphylactic reactions from food ingestion that occurred in China, 63 (17.3%) were caused by insects, particularly locusts and grasshoppers [50]. In another study conducted over two years at a Thai tertiary care hospital, 7 out of 36 (19.4%) cases of anaphylaxis from food were attributable to insect intake, with locusts and grasshoppers being more prevalent [51]. Regarding diagnosis, clinical cases are reported in which skin allergy testing was performed using commercial extracts of dried or fresh insects [45]. Regarding blood tests, ImmunoCAPs (Thermofisher®, Phadia®) containing whole insects, including cockroaches and silkworms, are available [45]. A recently acquired method of diagnosis is the Allergy Explorer (ALEX®), an in vitro diagnostic test for allergen-specific IgE (sIgE) assay, which recognizes the possible presence of cricket (Ach d), mealworm (Ten m) and locust (Loc m) molecules [52]. Therapeutic management is the same as for food allergies. There have been no reported cases of allergen-specific immunotherapy for food allergy to insects [45, 53]. With the marketing of numerous foods containing insects, it is to be expected that allergies to edible insects will also occur in Western countries, and therefore, an improvement and standardization of allergy diagnosis procedures is desirable to optimize their management.

**Table 1:** Epidemiology and diagnosis related to the main imported allergens in Italy

Allergens	Epidemiology	Diagnosis
**Ambrosia artemisiifolia L. (common ragweed)**	Accidental diffusion through air currents. Place of origin: North America. Place of invasion: Lombardy	Clinical history, Skin testing, blood testing
**Artemisia annua, Artemisia verlotiorum**	Accidental diffusion through air currents. Place of origin: China. Place of invasion: Trentino-South Tyrol	Clinical history, Skin testing, blood testing
**Cannabis Sativa**	Trade	Clinical history Skin Testing
**Imported food allergens from ethnic food**	Trade	Clinical history; Skin testing, blood testing (if available), Oral food challenge
**Edible Insects**	Trade	Clinical history; Skin testing, blood testing (if available), oral food challenge
**Asian Wasp Velutina, Solenopsis Invicta**	Trade, absence of natural enemies in the destination ecosystem	Clinical history; skin testing, blood testing (if available)

## Hymenoptera venom allergy

The combination of climate change, accidental introduction through commercial air traffic, and the absence of natural enemies in the target ecosystem has caused the appearance of a specific Hymenoptera, the *Asian Wasp Velutina* or *Asian Hornet*, in Europe and Italy [54, 55]. This type of Hymenoptera is native to India, China and Indonesia. In 2003, it was found in South Korea, and in 2004, in Europe, specifically in France. Subsequently, given its significant migration speed (18.3 ± 3.3 km per year), *Vespa velutina* spread to Spain, Portugal and the Netherlands and reached Italy in 2012. The first region to be reached was Liguria. Subsequently, its presence has been reported in northern and central Italy [56]. *Vespa velutina* poses a risk to both bio-vegetal diversity and human health. Specifically, the diet of its larvae is based on bees, which are decimated and cannot carry out their pollinator action [57]. In addition, it can inflict dangerous and often lethal stings to humans, with the possibility of complications, especially in the kidney and eye and, in rare cases, even anaphylaxis [55, 58, 59]. The venom of *Vespa velutina* contains proteins that could act as toxins and allergens. Allergen-wise, Vesp v 5 (antigen 5) is the dominant allergen; Vesp v1 (phospholipase A1) represents the minor allergen. No allergen-specific immunotherapy exists for patients with wasp velutin anaphylaxis [60]. Given the antigenic similarity, extracts of the venom of Vespula spp have been used to treat patients with Vespa velutin anaphylaxis [60]. In any case, preventive measures and action plans for allergic reactions are also important [61]. Another invasive alien imported fire ant (Solenopsis Invicta) was recently documented in Sicily [62]. It comes from South America, and its stings are related to severe allergic reactions [63]. 

## Conclusions

External events attributable to human action, such as climate change and the introduction non-native plants, foods, and Hymenoptera through trade, have contributed to the problem of allergen imports (Table 1). The consequence of this event is that some pediatric allergological fields considered acquired and stable in knowledge are changing over time. The pediatric allergist has the task of learning about imported allergens and the signs and symptoms by which they may manifest in the allergic individual. This preliminary step would allow to use proper diagnostic tests (skin testing, blood testing, challenge test) in order to identify the allergic individual’s sensitivity so that therapeutic interventions can be best directed.

### Electronic supplementary material

Below is the link to the electronic supplementary material.

Supplementary Material 1

## Data Availability

Not applicable.
