# Supplementary material for: Imported allergens in Italy: an emerging issue
Source: Ital J Pediatr. 2024 Mar 3;50:36. doi: 10.1186/s13052-024-01595-z (PMC10910788; doi:10.1186/s13052-024-01595-z)
Supplement: Supplementary file 1 — Supplementary Material 1 [file 13052_2024_1595_MOESM1_ESM.docx]

Dear Editor,
We thank you again for the opportunity offered to revise our article.

**Reviewer #1**

Dear Authors, thanks for review of your paper. Your document represents an interesting and actual work on a topic particularly relevant for the allergist. Reading is smooth and the provided informations are very useful for identifying emerging allergies. Just one note: I would propose integrating the text with a table of the allergens listed, their epidemiological characteristics and, where feasible, the execution of in vivo and in vitro diagnostic tests, in order to simplify reading.

I thank the reviewer for the suggestion. I have performed a supplementary table. I have added (if the reviewers agree) a new imported allergen documented very recently in Sicily during the time of the revision.


**Reviewer #2**

Thanks to the authors for highlighting an emerging and important problem like this.
Even the allergy to inhalants and hymenoptera which we considered acquired and stable from the point of view of knowledge is changing.
The challenge that the modern allergist will certainly have to face is demanding considering the diagnostic difficulties in particular in the food sector. The medical history certainly acquires more and more value but molecular diagnostic supports are obviously necessary to evaluate these uncommon and often hidden allergens.

I thank the reviewer.

I thank the reviewer for the time and efforts he/she dedicated to our article.

I hope that the revised manuscript will be suitable for publication.

Thanks for your attention.

Regards.
